# Early Detection of Alzheimer’s Disease Using Non-invasive Near-Infrared Spectroscopy

**DOI:** 10.3389/fnagi.2018.00366

**Published:** 2018-11-09

**Authors:** Rihui Li, Guoxing Rui, Wei Chen, Sheng Li, Paul E. Schulz, Yingchun Zhang

**Affiliations:** ^1^Department of Biomedical Engineering, University of Houston, Houston, TX, United States; ^2^Guangdong Provincial Work Injury Rehabilitation Hospital, Guangzhou, China; ^3^Nanjing Ruihaibo Medical Rehabilitation Center, Nanjing, China; ^4^Department of Physical Medicine and Rehabilitation, The University of Texas Health Science Center at Houston, Houston, TX, United States; ^5^Department of Neurology, The University of Texas Health Science Center at Houston, Houston, TX, United States

**Keywords:** mild cognitive impairment, Alzheimer’s disease, functional near-infrared spectroscopy, hemodynamic response, oxygenated hemoglobin

## Abstract

Mild cognitive impairment (MCI) is a cognitive disorder characterized by memory impairment, wherein patients have an increased likelihood of developing Alzheimer’s disease (AD). The classification of MCI and different AD stages is therefore fundamental for understanding and treating the disease. This study aimed to comprehensively investigate the hemodynamic response patterns among various subject groups. Functional near-infrared spectroscopy (fNIRS) was employed to measure signals from the frontal and bilateral parietal cortices of healthy controls (*n* = 8), patients with MCI (*n* = 9), mild (*n* = 6), and moderate/severe AD (*n* = 7) during a digit verbal span task (DVST). The concentration changes of oxygenated hemoglobin (HbO) in various subject groups were thoroughly explored and tested. Result revealed that abnormal patterns of hemodynamic response were observed across all subject groups. Greater and steeper reductions in HbO concentration were consistently observed across all regions of interest (ROIs) as disease severity developed from MCI to moderate/severe AD. Furthermore, all the fNIRS-derived indexes were found to be significantly and positively correlated to the clinical scores in all ROIs (*R* ≥ 0.4, *P* < 0.05). These findings demonstrate the feasibility of utilizing fNIRS for the early detection of AD, suggesting that fNIRS-based approaches hold great promise for exploring the mechanisms underlying the progression of AD.

## Introduction

Alzheimer’s disease (AD) is a chronic progressive neurodegenerative brain disease that typically presents as a slight failure of memory that gradually becomes more acute (Kumar et al., 2015). In recent years, AD has been considered the most common cause of dementia, accounting for 60–80% of dementia cases around the world (Assoc, 2015). As previous studies have demonstrated, AD is characterized by the presence of pathological amyloid-beta deposits and neurofibrillary tangles (hyperphosphorylated tau protein aggregates), along with a significant loss of neurons and deficits in the neurotransmitter systems (Cohen and Klunk, 2014; Palmqvist et al., 2015). Based on this evidence, it was hypothesized that these proteins may disrupt the communication among nerve cells and damage the cells, leading to the development of AD. The specific role of these proteins, however, remains unknown and requires further validation.

Patients with AD generally suffer from multiple functional impairments, including deficits to memory, reasoning, communication, and behavioral function that interfere with the individual’s daily life (Karantzoulis and Galvin, 2011). While there is currently no cure for AD, medication therapy or daily training have been able to alleviate the progression of the disease and improve the subject’s quality of life (Gates and Sachdev, 2014; Folch et al., 2016). As the AD pathology is now suspected to start long before the symptoms begin, diagnosis of the disease at earlier stages is of great clinical importance. Amnestic mild cognitive impairment (aMCI) is an intermediate state of impairment characterized by a cognitive decline that exceeds that of normal aging (Petersen et al., 2001). Although it does not meet the clinical criteria for AD (Petersen, 2009), patients with aMCI are more likely to develop AD (Petersen, 2004). With this in mind, there is an urgent need to develop an approach for the identification of individuals before or during the earliest stages of AD, with the hope that intervention could delay or perhaps even prevent the onset of clinical symptoms. Additionally, approaches that are able to monitor the stages of AD can be used to evaluate the efficacy of potential medication therapies during the treatment of AD.

Currently, the detection and diagnosis of AD relies primarily on patients’ medical history, laboratory neuropsychological examinations, and clinical rating score, such as Mini-Mental State Examination (MMSE) or Clinical Dementia Rating (CDR) (Folstein et al., 1975; Morris, 1993). Unfortunately, clinical evaluation requires experienced clinicians and exhaustive testing sessions, which not only incurs additional medical expenditures but also introduces subjectivity and variability to the diagnostic outcome. To address this, brain imaging techniques – especially magnetic resonance imaging (MRI) and positron emission tomography (PET) – have been introduced to the field in recent years. MRI is generally utilized as a structural and functional imaging technique to visualize AD-linked cortical atrophy and to capture the change of brain connectivity (Jack et al., 1997; Wang et al., 2006; Scheltens and van de Pol, 2012). It has been suggested that the volume of medial temporal lobe (MTL) is significantly smaller in AD patients than control subjects (Jack et al., 1997). Functional MRI (fMRI) study also found that functional connectivity between the right hippocampus and a set of regions was disrupted in AD (Wang et al., 2006). Conversely, PET provides a reliable approach to capture the metabolic activity associated with the amyloid plaques that are characteristic of AD (Forsberg et al., 2008; Kadir et al., 2012). Recently, the development of advanced PET tracers, such as Pittsburgh Compound-B (PiB) and Fluoro-2-deoxy-D-glucose (FDG), have made *in vivo* imaging of the amyloid plaques possible, with striking differences observed between the healthy controls and AD subjects in both amyloid deposition and cerebral glucose metabolism (Cohen and Klunk, 2014). Despite their benefits, these techniques are costly to perform and have rigorous measurement requirements as well as high cost expense, preventing their use as routine clinical tools.

Functional near-infrared spectroscopy (fNIRS) is a developing optical imaging technology that has been increasingly employed in the neuroimaging field over the past 20 years. It utilizes near infrared lights at two distinct wavelengths (between 600 and 1000 nm) to measure the concentration changes of oxygenated hemoglobin (HbO) and deoxygenated hemoglobin (HbR) associated with the metabolic activity of neurons in the outer layers of the cortex (Ferrari and Quaresima, 2012; Boas et al., 2014; Li et al., 2017). Previous studies have found a high correlation between the hemodynamic response measured by fNIRS and the blood oxygen level dependent (BOLD) response obtained by fMRI, making the two modalities as rough analogs (Cui et al., 2011; Sato et al., 2013). fNIRS, however, offers multiple benefits over fMRI and PET; it is non-invasive and features a higher temporal resolution, high portability, lower cost, lower susceptibility to movement artifacts, lack of ionizing radiation, and poses less constraint to subjects during the measurement (Ehlis et al., 2014). Additionally, fNIRS provides functional imaging by measuring cortical hemodynamic responses. These benefits make fNIRS a potential alternative technique to fMRI and PET for the convenient and non-invasive diagnosis or therapeutic monitoring in AD patients in clinic.

A number of studies have examined the feasibility of using fNIRS to compare hemodynamic responses in healthy controls and AD patients (Hock et al., 1997; Arai et al., 2006; Herrmann et al., 2008; Zeller et al., 2010; Araki et al., 2014; Uemura et al., 2016; Beishon et al., 2017; Vermeij et al., 2017). The results of these projects show that AD patients exhibited lower levels of activation at specific brain regions when compared to healthy controls during various cognitive tasks. Hock et al. (1997) reported a pronounced decrease in HbO and total hemoglobin (HbT) in the parietal cortex of AD patients when given a verbal fluency task (VFT). Another study, including healthy, MCI, and AD groups, revealed that HbO concentrations in the frontal and bilateral parietal areas were significantly reduced in the AD group, whereas the activation in the MCI group was significantly lower only in the right parietal area (Arai et al., 2006). These results suggest that fNIRS might have the potential to detect the AD, even at early stages. In addition, fNIRS has also been employed to monitor the effect of pharmaceutical intervention on AD patients. In particular, Araki et al. (2014) used fNIRS to monitor the effect of memantine on AD patients and reported a significant difference in cerebral blood flow between the treatment group and control group. This suggests that fNIRS might be able to effectively monitor AD treatment in clinic.

Although the previous results support the potential applications of fNIRS to AD detection, there are multiple issues which remain unresolved and need to be addressed. First, although most fNIRS studies reported significant differences between healthy and AD groups, how well the technique is able to characterize differences across the various stages of AD remains unknown. Investigating this requires the recruitment of a number of subjects at various stages of AD, along with MCI patients, to provide comprehensive comparisons and evaluate the effectiveness of fNIRS for diagnosis and/or therapeutic monitoring. Secondly, the hemodynamic response associated with the mechanisms of AD progression has not yet been thoroughly investigated and documented. It is expected that a more intact experimental design, incorporating advanced fNIRS-derived metrics to investigate multiple subjects groups across several brain regions, may help explore the pathological mechanisms of AD development.

To address above challenges, the present study sought to comprehensively investigate the hemodynamic responses of healthy subjects, patients with MCI and varying degrees of AD. Subjects were recruited and divided into four groups – the healthy control group (HC), the aMCI group (MCI), the mild AD group (MAD), and the moderate/severe AD group (MSAD). A digit verbal span task (DVST) was employed as a cognitive task, and fNIRS signals were measured from the frontal area and bilateral parietal areas. We hypothesized that a certain pattern related to presence of aMCI and the severity of AD can be observed among four different groups by the proposed fNIRS-derived metrics. It is further hypothesized that the observed pattern will show characteristics that vary with the type, and severity of the disease.

## Materials and Methods

### Participants

In this study, 22 patients were recruited from a local hospital, along with eight age and education-matched healthy volunteers from the local community. All subjects and controls were right-handed and had no history of cerebrovascular lesions or psychiatric disorder. No subject had any previous experience with the experimental task. Subjects were subsequently divided into four groups according to medical examination by a psychiatrist: the HC group (HC, 63.6 ± 6.5 years, 6M/2F), MCI group (MCI, 70.3 ± 5.4 years, 6M/3F), MAD (MAD, 72.5 ± 7.3 years, 2M/4F), and MSAD group (MSAD,76 ± 4.8 years, 3M/4F). In addition, the mental state of each subject was examined using the MMSE, a 30-point questionnaire providing a quantitative measure of cognitive status or impairment (Folstein et al., 1975), and scores were recorded. The demographic information for all subjects, including age, gender and MMSE scores, are summarized and shown in Table 1. The experiment was approved by the Research Ethics Board of Guangdong Provincial Work Injury Rehabilitation Center and performed in accordance with the Declaration of Helsinki. Each subject was fully informed about the purpose of the research and provided written, informed consent prior to the start of the experiment.

**Table 1:** The demographic information of all subjects.

Characteristic	HC (*n* = 8)	MCI (*n* = 9)	MAD (*n* = 6)	MSAD (*n* = 7)
Ages (years)	63.6 ± 6.5	70.3 ± 5.4	72.5 ± 7.3	76 ± 4.8
Gender (M/F)	6M/2F	6M/3F	2M/4F	3M/4F
MMSE	28.2 ± 2.2	26 ± 2.2	19.7 ± 3	9.4 ± 1.7

### Digit Verbal Span Task

**FIGURE 1:**
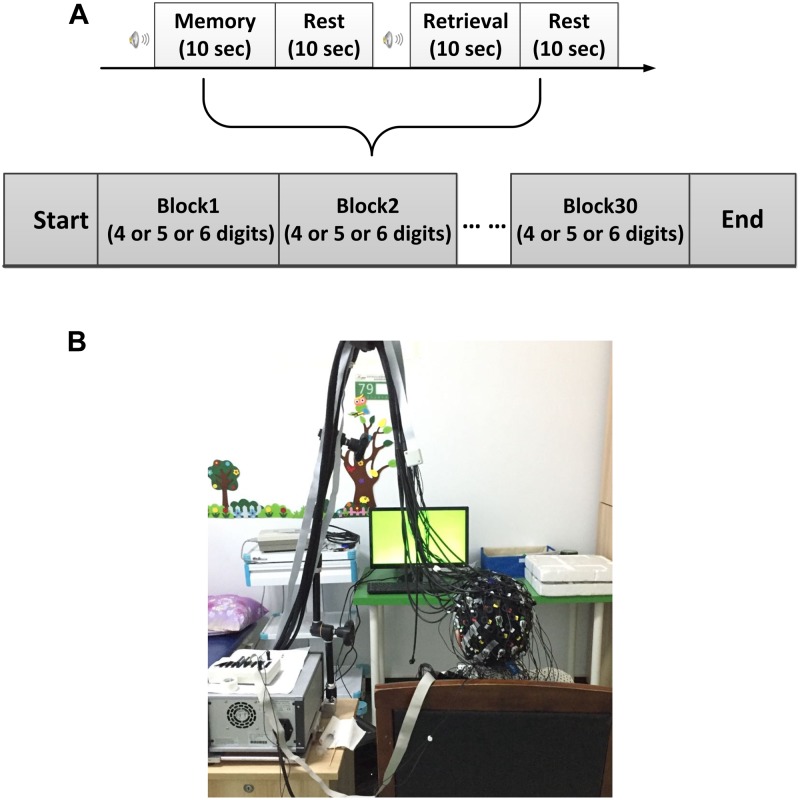
Experimental design. **(A)** The digit verbal span task used in this study. **(B)** Illustration of experimental environment.

All experiments were performed in a confined room to reduce any environmental disturbance. Before beginning the experimental task, subjects were seated in a comfortable chair and asked to rest with eyes closed for 3 min, during which a baseline fNIRS measurement was acquired. Subjects then underwent a DVST consisting of 30 individual blocks (Figure 1A). Each block started with a encoding task, during which subjects were asked to memorize a number sequence that was displayed on a PC screen 1.5 m in front of them (Figure 1B). Number sequences varied in length from 4 to 6 digits (individual digits ranged from 1 to 9; 10 blocks for each number length) and were presented for 10 s before disappearing. Encoding was followed by a 10-s resting period, during which the subject was asked to stay relaxed. Afterward, subjects were instructed to verbally recall the number and responses were recorded for performance evaluation. To minimize the subject-expectancy effect, all 30 sequences were unique and randomly displayed to the subjects during the experiment. Before the beginning of the experiment, detailed explanations were given regarding the task and participants were allowed sufficient practice to become familiar with the experimental procedures.

### fNIRS Configuration

**FIGURE 2:**
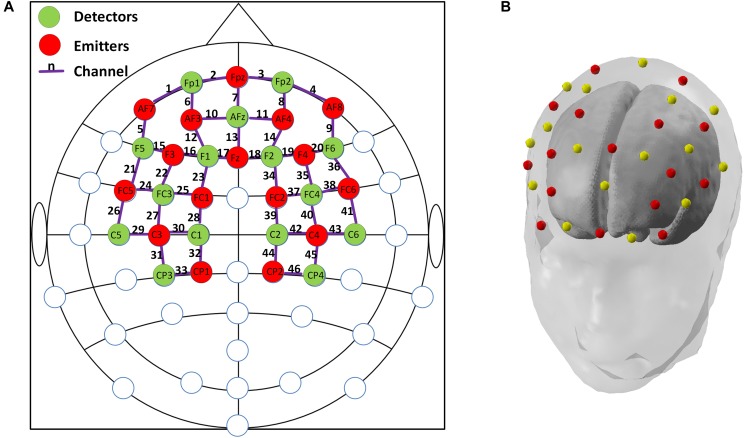
**(A)** The configuration of fNIRS optodes and channels. Red circles denote the emitters, green circles denote the detectors, the purple lines and numbers are defined as fNIRS channels. Labels within circles denote the locations in international 10–20 EEG placement. Frontal area: channels 1–20; Left parietal: channels 21–33; and Right parietal: channels 34–46; **(B)**. A demonstration of the standard 3-D head model with the emitters (red dots) and detectors (yellow dots) generated by nirsLAB (NIRx Medizintechnik GmbH, Germany).

In this study, a multi-channel NIRScout system (NIRx Medizintechnik GmbH, Germany) with 15 emitters and 16 detectors was used to measure the fNIRS signals at a sampling rate of 3.91 Hz. The wavelengths used for oxy- and deoxy-hemoglobin detection were 760 and 850 nm, respectively. Inter-optode distances were fixed at ∼3 cm and a total of 46 measurement channels were equidistantly distributed throughout the frontal and bilateral parietal areas, according to the international 10–20 Electroencephalography (EEG) placement system. An fNIRS channel was defined as an emitter-detector pair, and all emitters and detectors were mounted to an elastic cap using plastic grommets and holders, which ensured that optodes made good contact with the subject’s head. A schematic illustration of the fNIRS channel locations is shown in Figure 2.

### Data Preprocessing

The fNIRS data were processed and analyzed for each individual subject using MATLAB (2014a, MathWorks, Natick, Massachusetts). Each fNIRS channel was visually inspected and channels with large spikes were marked as “noisy” and excluded from further analysis. To process the fNIRS signal, a 4th order Butterworth band-pass filter with cut-off frequencies of 0.01–0.2 Hz was applied to remove artifacts, including cardiac interference (0.8 Hz) and respiration (0.2–0.3 Hz) (Zhang et al., 2005). The concentration changes of hemoglobin (HbO and HbR) were computed according to the Modified Beer-Lambert Law [the differential path length factors for the higher (850 nm) and lower (760 nm) wavelengths were 6.38 and 7.15, respectively] (Scholkmann et al., 2014). It is worthy of note that data analysis focused solely on HbO signals in this study. This is because HbO signal is considered to be a more robust and sensitive fNIRS parameter than HbR to task-associated changes, and shows stronger correlation with fMRI BOLD response (Plichta et al., 2006; Sato et al., 2006; Cui et al., 2011). In addition, HbO have been previously reported as a more preferable measure in characterizing the aging effect in relevant aging studies (Vermeij et al., 2014; Yap et al., 2017).

For each cleaned fNIRS channel, HbO signal in each block was segmented, resulting in 30 trials of HbO signals with 20-s interval beginning at the onset of the encoding task. Then all segmented HbO signals were averaged and a grand-averaged HbO signal was obtained for each channel and each subject. Subsequent analysis was performed using these averaged HbO signals.

### Data Analysis

Three brain regions, including the frontal, left parietal, and right parietal regions, were of interest in this study according to previous studies that showed meaningful findings in MCI/AD cohort (Hock et al., 1997; Arai et al., 2006), we therefore manually assigned a fixed number of fNIRS channels to the corresponding areas, as indicated in Figure 2. Although previous studies have adopted various active channel selection strategies to opt for activated channels, such as *t*-values-based approach and amplitude-based approach (Khan and Hong, 2017; Hong et al., 2018), the abnormal hemodynamic responses from four different groups that were incompatible with canonical hemodynamic response made these strategies unsuitable in this study. Therefore, we averaged the HbO signals from all cleaned fNIRS channels within the same brain region, resulting in a single, representative HbO signal for each region of interest (ROI) of each individual subject.

Based on above preprocessing methods, two parameters – mean change of HbO concentration (MHbO) and the slope of the HbO change (SPHbO) – were derived for each of the four groups. As suggested by previous studies, change in HbO concentration generally indicates the metabolic activity of local neurons, which is tied to functional activity and may take more than 10-sec to reach a significant activation level (Khan et al., 2014; Kato et al., 2017). We therefore computed the MHbO from each brain region using a 3–12 s window. The SPHbO, on the other hand, was defined as the linear change rate for the HbO signal to reach its peak or nadir within 10 s starting from the task onset, which reflected how the specific brain region responded to the given task. To estimate the SPHbO, we first identified the time point at which absolute value of the HbO signal peaked, and then a simple linear regression was performed to estimate the slope between the task onset and identified peak. The SPHbO in each ROI was then obtained as the coefficient of the linear regression model.

### Statistical Analysis

The differences among all the four groups in terms of the MMSE scores and task performance were evaluated using two-sample *t*-tests with a Bonferroni-Holm multi-comparison correction (4 groups, 6 comparisons in total) (McLaughlin and Sainani, 2014). Task performance for each subject was defined as the number of correct sequences he/she recalled in the retrieval task, up to a maximum of 30. Briefly, the two-sample *t*-test was performed between any two groups, resulting in six *p*-values for all comparisons. Then the Bonferroni-Holm multi-comparison correction was performed. We first ordered all *p*-values from smallest to greatest (*p_i_, i* = 1,2…6), the corrected *p*-value of *ith* comparison (${p^{\prime }}_{i}$) was given by:

$${p^{\prime }}_{i}=\frac{p_{\text{target}}}{n-i+1}$$

where *p*_target_ denotes the target significant level in the test, which was set to 0.05 in this study, *n* denotes the total number of comparisons in this study. We considered the difference of *ith* comparison was significant if *p_i_* is less than ${p^{\prime }}_{i}$.

fNIRS-derived indexes, including the MHbO and SPHbO for each brain region, were similarly assessed using two-sample *t*-tests with a Bonferroni-Holm multi-comparison correction (4 groups, 6 comparisons in total). Finally, to evaluate the association between hemodynamic signals and clinical rating scores, Pearson’s correlation coefficients between the MMSE scores and proposed fNIRS-derived indexes were computed with a significance threshold of 0.05.

## Results

### Behavioral Analysis

**FIGURE 3:**
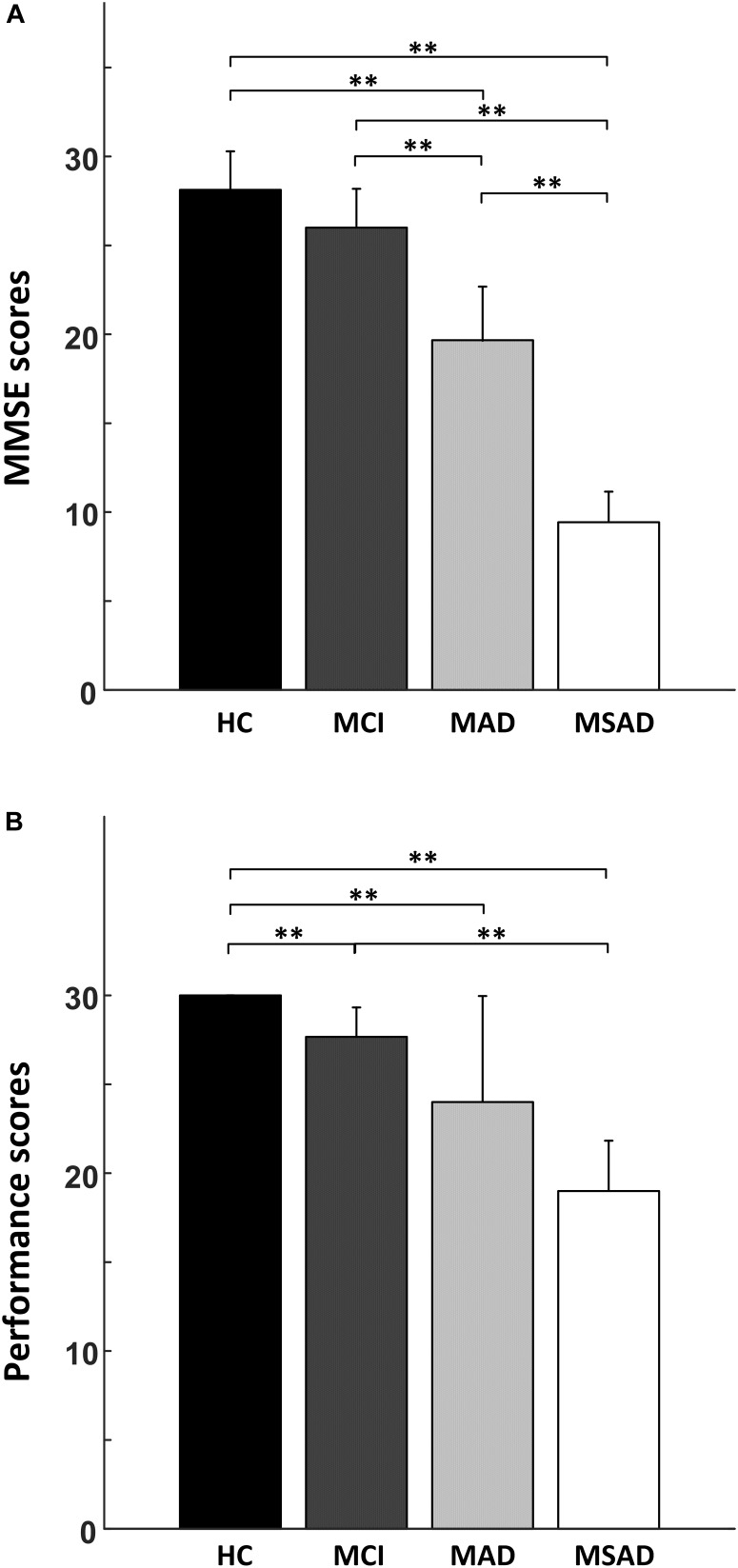
The statistical analysis results of the behavioral measures. **(A)** The comparison of MMSE scores between all four groups; **(B)** The comparison of task performance between all groups. “^∗∗^” symbol denotes significant differences after Bonferroni-Holm correction.

The comparisons between four groups in terms of the MMSE scores and task performance are shown in Figure 3 and summarized in Table 2. Briefly, MMSE scores showed a negative correlation with the severity of AD and statistical analysis identified significant differences between each of the groups (*p* < 0.001), with the single exception of the MMSE scores between healthy controls and MCI group, which were not significantly different after multiple comparisons correction (Figure 3A). For task performance, a similar negative correlation was found with the severity of AD, as shown in Figure 3B. Statistical analysis identified significant differences between the performance scores of each condition, with the exception of comparisons between MCI and MAD, MAD and MSAD, which were not significantly different.

**Table 2:** Summary of the statistical analysis results.

Characteristics		*p*-value of two sample *t*-test					
		HC vs. MCI	HC vs. MAD	HC vs. MSAD	MCI vs. MAD	MCI vs. MSAD	MCI vs. MSAD
MMSE		0.063	<0.001^∗∗^	<0.001^∗∗^	<0.001^∗∗^	<0.001^∗∗^	<0.001^∗∗^
Performance		0.001^∗∗^	0.013^∗∗^	<0.001^∗∗^	0.099	<0.001^∗∗^	0.073
MHbO	FA	0.048^∗^	<0.001^∗∗^	<0.001^∗∗^	0.144	0.047^∗^	0.397
	LP	0.069	0.019^∗^	0.021^∗^	0.057	0.751	0.738
	RP	0.082	<0.001^∗∗^	0.035^∗^	0.521	0.632	0.957
SPHbO	FA	0.021^∗∗^	0.002^∗∗^	<0.001^∗∗^	0.086	0.005^∗∗^	0.075
	LP	0.056	0.009^∗∗^	0.002^∗∗^	0.689	0.078	0.075
	RP	0.016^∗^	0.002^∗∗^	<0.001^∗∗^	0.319	0.031^∗^	0.127

*“^∗∗^” denotes a significant difference with Bonferroni-Holm correction, while “^∗^” denotes significant difference without Bonferroni-Holm correction. FA: Frontal area; LP: Left parietal; and RP: Right parietal.*

### Result of fNIRS Data

**FIGURE 4:**
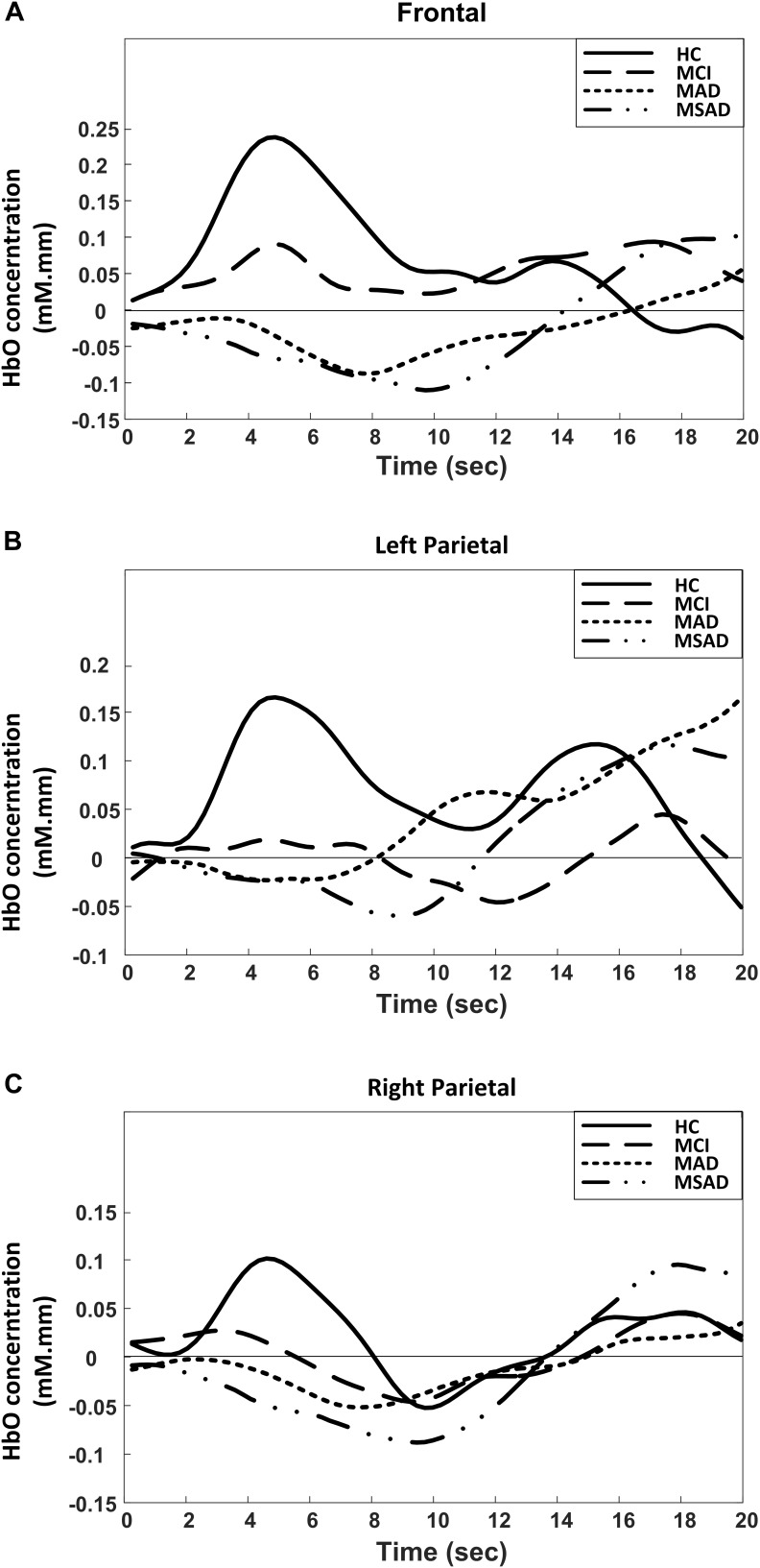
Group-averaged changes of HbO concentration for all participants in three brain regions during encoding task. **(A)** Frontal area; **(B)** Left parietal; **(C)** Right parietal.

The group analysis results of the HbO change with respect to subject group and cortical region are summarized and shown in Figure 4. As the results demonstrate, unique patterns of HbO fluctuation during the encoding task could be associated with the different groups in all three brain regions. In particular, the activation pattern of HbO in HC group tended to be a normal hemodynamic response – it appeared as a rapid increase in the HbO concentration that gradually returned to the baseline. On the contrary, the hemodynamic response in the MCI group showed a mild and delayed rise in HbO concentration during the encoding task, while the AD groups (MAD and MSAD) showed an apparent decrease and delay in the HbO activation.

**FIGURE 5:**
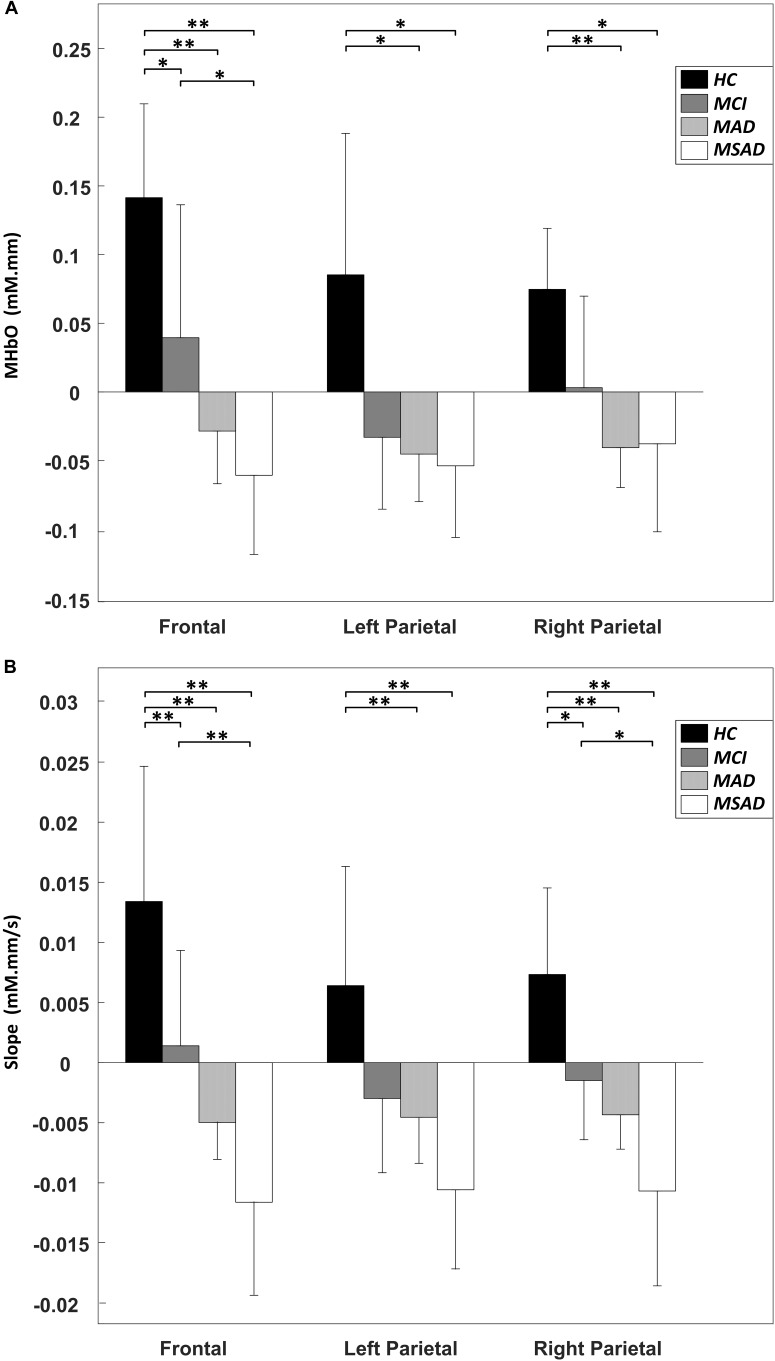
The statistical summary of fNIRS data. **(A)** The mean change of HbO concentration (MHbO) in three brain regions among all groups. **(B)** The mean slope of HbO concentration change (SPHbO) in three brain regions among all groups. “^∗∗^” denotes a significant difference with Bonferroni-Holm correction, while “^∗^” denotes a significant difference without Bonferroni-Holm correction.

The MHbO values of all three brain regions were computed from individual subject data and are summarized in Figure 5A. The HC group showed the strongest HbO activation across all ROIs, while the MCI group showed a mild increase or decrease in HbO in each of the ROIs. For AD groups, including MAD and MSAD, noticeable declines in HbO concentration were observed in all ROIs, with the MSAD group showing the greatest reduction. Statistical analysis suggested that, when no multiple comparison correction was applied, there were significant differences between the HC and all other three groups in frontal area, while there were only significant differences between the HC and AD groups in bilateral parietal areas. These differences, however, were only observed between HC and AD groups in frontal area after a Bonferroni-Holm correction was applied (Figure 5A).

Figure 5B shows the mean SPHbO values for each brain regions. The HC group showed a steep upward trend in HbO change after the task onset in all ROIs, while the MCI group showed only mild rate of HbO change after task onset and found a positive SPHbO only in the frontal region. For AD groups, including MAD and MSAD, apparent negative HbO changes were observed in all brain regions after the task onset. In particular, the rate of HbO reduction was steeper in the more severe AD group. Similarly, statistical analysis suggested that, even with Bonferroni-Holm correction, there were significant differences between the hemodynamic responses of the HC and MCI/AD groups in the frontal area, while significant differences were only observed between the HC and MAD/MSAD groups in bilateral parietal areas. Additionally, a significant difference between MCI group and MSAD group was found in the frontal area. Results of the statistical analysis on the MHbO and SPHbO measurements are summarized in Table 2.

**FIGURE 6:**
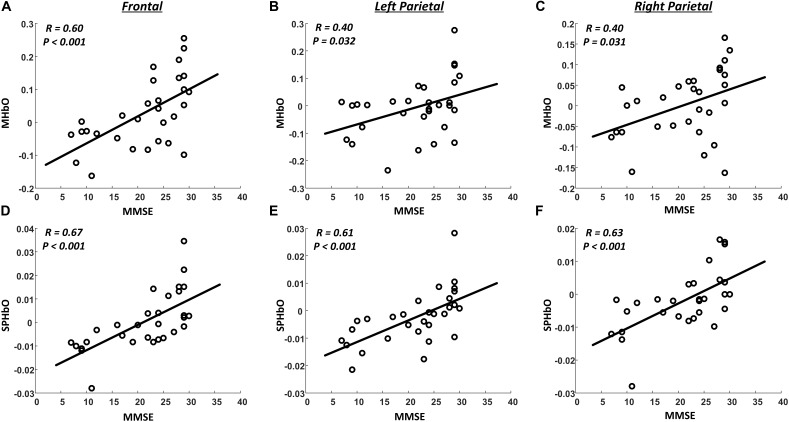
Summary of correlation analysis results. The scatter plots (dots) denote the relationship between MMSE scores and fNIRS-derived indexes from all subjects (**A–C**: MHbO; **D–F**: SPHbO). The lines are linear regression lines between MMSE scores and fNIRS-derived indexes.

We also evaluated, using correlation analysis and linear regression analysis, the relationship between fNIRS-derived indexes (MHbO and SPHbO) and MMSE scores. As illustrated in Figure 6, all the fNIRS-derived indexes, including MHbO and SPHbO, were found to be significantly and positively correlated to the MMSE scores in all brain regions (*R* ≥ 0.4, *P* < 0.05). These results demonstrated that the hemodynamic response detected by fNIRS could serve as a sensitive tool for the evaluation of cognitive function in clinic.

## Discussion

The goal of this study was to evaluate fNIRS as a potential tool for the early detection, functional differentiation, and clinical monitoring of MCI, MAD, and MSAD. The main contributions of the present study are, on one hand, a comprehensive investigation of hemodynamic properties covering healthy controls and patients at various stages of cognition impairment was performed. By measuring fNIRS signals from the frontal and parietal regions during a simple DVST, we were able to consistently show different patterns of HbO response among four subject groups. On the other hand, the fNIRS-derived indexes, including MHbO and SPHbO, were significantly and positively correlated with clinical rating scores, demonstrating the feasibility of utilizing fNIRS as a routine tool in clinic to monitor the progression of AD.

As a neurodegeneration disease, AD-linked atrophy can cause the alterations in the anatomical structure and functional organization of the brain, affecting the metabolic activity of cortical neuron populations. These changes may also alter the optical properties of brain tissues, which can be detected via optical imaging techniques. Recently, fNIRS studies have attempted to characterize AD-linked tissue degeneration. The main findings of these studies reflected abnormal activities in the prefrontal and parietal cortices of MCI and AD patients during various cognitive tasks. Hock et al. (1997) reported a significant reduction of HbO in parietal area during a verbal fluent task in AD patients, while Herrmann et al. (2008) found that there was a reduction in the HbO response of the dorsolateral prefrontal cortex in patients with AD. In another fNIRS study, the HbO changes in the frontal and bilateral parietal areas of AD subjects were reported to be significantly decreased, while a significant decrease in HbO in the right parietal area alone was observed in MCI patients (Arai et al., 2006). In this study, we have sought to further explore and integrate the findings of literature. NIRS optodes were placed over the frontal and bilateral parietal areas, data was recorded from four recruited groups to provide a more comprehensive analysis of the progression of AD. Specifically, as shown in Figure 5, we found significant alterations in the regional MHbO and SPHbO measurements from the three patient groups when compared to healthy controls. Further regional hemodynamic differences were observed between the MCI and MSAD groups, suggesting the fNIRS has potential to monitor the disease progression from MCI to AD at various stages.

Two things should be noted here: (a) the statistical testing of MHbO between HC and MCI groups showed no significant difference when a Bonferroni-Holm correction was applied (Figure 5A); (b) the significant differences in MHbO between MCI and MAD, MAD and MSAD groups were not observed in this study even without Bonferroni-Holm correction. These may be partially explained by the limited sample size and large variance within each group, which led to a large amount of data overlap and relatively small group differences, as shown in Figure 5A. Future investigations that increase the number of subjects may be able to address the above issues and consolidate the findings in this study. Regardless, the immediate research concern is the early detection and monitoring of AD; it is more meaningful to differentiate between the HC, MCI, and AD groups, which has been largely achieved here (Figure 5).

Another finding of this study is that, by revealing the mean value of HbO (MHbO) and SPHbO, we quantitatively demonstrated the differences in the blood flow response patterns between healthy controls and different patient groups. As shown in Figure 4 and Figure 5A, statistical results suggest a uniform response pattern in frontal area, where increasingly significant reductions in HbO were observed as the disease severity developed from MCI to MSAD. This is in line with the findings from previous studies that HbO reductions in the frontal area were observed in MCI patients and AD patients (Arai et al., 2006; Herrmann et al., 2008; Uemura et al., 2016; Yap et al., 2017). In addition, based on the SPHbO patterns shown in Figure 4 and Figure 5B, the trend and velocity of HbO changes revealed a pattern of hemodynamic response, wherein HbO signals showed a greater declination rate and a longer recovery delay as the disease severity increased from MCI to severe AD. Two possible physiological mechanisms may be supported by these findings. On one hand, when performing a cognitive task, AD patients might require increased oxygen consumption due to functional impairments, which would lead to a greater reduction in HbO shortly after the task onset and the subsequent influx of a large amount of HbO, as shown in Figure 4. On the other hand, as cortical neurodegeneration caused by MCI or AD may induce functional reorganization, additional circuitry or brain areas may be needed to be recruited to support declining brain functions, which would again cause increased overall oxygen consumption (Park and Bischof, 2013). It should be noted that, as fNIRS is only able to measure the hemodynamic response on the cortical surface (1–3 cm), it is not possible to investigate any potential pathological changes to subcortical or deep structures that may cause the abnormal reduction and prolonged lag of the hemodynamic response in patient groups. Despite the limited depth resolution of fNIRS, however, our findings based on the extracted MHbO and SPHbO measurements have validated the great potential of fNIRS as a portable and reliable tool for the routine clinical study of AD. In particular, our results suggested that the frontal area could be considered a primary ROI for the study of AD, wherein MHbO and SPHbO may serve as valuable biomarkers for early detection or diagnosis.

As shown in Figure 3 and Table 2, the behavioral results, including the MMSE scores and performance of DVST, demonstrated a significant difference between HC and AD groups after Bonferroni-Holm correction. However, no significant difference in MMSE scores was observed between HC and MCI groups even without the Bonferroni-Holm correction. This indicates the limitations and challenges in differentiating MCI patients from healthy population using regular clinical rating scores. For example, although MMSE has been widely used in clinic and research findings can be easily compared, it is subject to ceiling effects that reduce sensitivity when distinguishing between HC and MCI patients (Tombaugh and Mcintyre, 1992). In contrast, floor effects also limit MMSE application to patients with more advanced AD. Results may be further influenced by subject age and education background, limiting their sensitivity and validity when evaluating disease progression or AD onset (Robert et al., 2010). In present study, we have demonstrated clear and distinguishable patterns of HbO response obtained from healthy controls and patient groups by applying a cognitive task in conjunction with fNIRS measurement. In particular, as shown in Figure 6, the correlation analysis results suggested significantly positive correlations between MMSE scores and all fNIRS-derived indexes (*R* ≥ 0.4). These findings therefore provide convinced evidence that the proposed fNIRS-based neuroimaging approach holds great promise as an advanced tool for the screening and monitoring of AD.

One of the main limitations of this study is only commonly used indexes were derived from the fNIRS signal to quantify the difference among four groups. Actually, previous studies have derived various features from fNIRS signal to represent the properties of hemodynamic response, such as variance, skewness, kurtosis, and initial dips (Santosa et al., 2014; Hong and Naseer, 2016; Hong and Santosa, 2016; Naseer et al., 2016; Hong et al., 2017; Zafar and Hong, 2017). Although the proposed indexes in this study were significantly correlated with clinical rating scores, more effort should be taken to optimize the approach in characterizing the difference among all groups, including evaluating various features and classification techniques in a future work. Furthermore, it should be recognized that the cognitive task used to evoke cortical responses and subject sample sizes may play essential roles in cognitive studies. The present study employed the DVST paradigm, as opposed to the VFT that has been used in some recent AD studies (Arai et al., 2006; Herrmann et al., 2008; Yap et al., 2017). The DVST was considered easier and more acceptable for the patients with severe AD, making it a preferable choice for this investigation. The significant results of this study would support this use for the DVST, though further testing with other paradigms may be necessary to fully characterize patients’ hemodynamic response. Additionally, despite presenting clear patterns and differences between conditions, the limited sample size of this paper may have been insufficient to characterize more subtle trends between the patient groups. Therefore, increasing the sample size will be needed in future work.

## Conclusion

This is the first study to thoroughly characterize the hemodynamic responses in the frontal and bilateral parietal areas of healthy controls, MCI patients, and two AD groups using fNIRS techniques in conjunction with a DVST. The greatest reduction (MHbO) and steepest decline (SPHbO) in HbO concentration were observed in the MSAD group, followed by MAD and MCI groups. Significant differences in the MHbO between the HC group and all patient groups were mainly confirmed in the frontal area, while significant differences in SPHbO between the HC group and all patient groups were found in each of the ROIs with Bonferroni-Holm correction. While improvement in the sample size and experimental protocol should be considered in the future, the findings of this study provide evidence that fNIRS could serve as a potential tool for the early detection and screening of AD progression.

## Author Contributions

RL, PS, and YZ designed the study. WC, and GR acquired the data. RL, SL, and PS analyzed and interpreted the data. RL, GR, and YZ drafted the manuscript. RL, GR, SL, PS, and YZ approved the final manuscript.

## Conflict of Interest Statement

The authors declare that the research was conducted in the absence of any commercial or financial relationships that could be construed as a potential conflict of interest.
